# A novel p70 S6 kinase-microRNA biogenesis axis mediates multicellular spheroid formation in ovarian cancer progression

**DOI:** 10.18632/oncotarget.9345

**Published:** 2016-05-13

**Authors:** Sophia So Ngo Lam, Carman Ka Man Ip, Abby Sin Chi Mak, Alice Sze Tsai Wong

**Affiliations:** ^1^ School of Biological Sciences, University of Hong Kong, Hong Kong

**Keywords:** p70 S6 kinase, microRNA biogenesis, multicellular spheroid formation, ovarian cancer

## Abstract

Ovarian cancer is the leading cause of death of all gynecologic tumors, associated with widespread peritoneal dissemination and malignant ascites. Key to this is the ability to form multicellular spheroids (MCS); however, the tumor-specific factors that regulate MCS formation are unclear. p70 S6 kinase (p70^S6K^), which is a downstream effector of phosphatidylinositol 3-kinase/Akt, is frequently constitutively active in ovarian carcinoma. Here we identify p70^S6K^ as a vital regulator of MCS formation. We also uncover a new mechanism of p70^S6K^ function as a component of the microRNA biogenesis machinery in this process. We show that p70^S6K^ phosphorylates, and inhibits the interaction of tristetraprolin (TTP) and Dicer that promotes the expression of a subset of miRNAs, including the maturation of miR-145. Twist and Sox9 are two divergent targets of miR-145, thereby enhancing N-cadherin, but not other cadherin, expression and MCS formation. Activating miR-145 suppresses ovarian tumor growth and metastasis in an orthotopic xenograft mouse model. Meta-analysis in the Oncomine database reveals that high p70^S6K^ and low TTP levels are associated with ovarian tumor progression. These results define a critical link between p70^S6K^, miRNA maturation, and MCS formation that may underlie poor clinical outcome of ovarian cancer patients for developing novel therapeutic strategies.

## INTRODUCTION

Ovarian cancer is the leading cause of death from gynecologic cancers [1]. The majority of cases (70%) are diagnosed after the disease has already metastasized with widespread peritoneal dissemination and malignant ascites. Several lines of evidence suggest that multicellular spheroids (MCS) play an important role in the biology of ovarian cancer. First, ovarian cancer cells in peritoneal ascites exist as MCS (30-200 μm) [2]. Second, MCS can readily attach to and disaggregate on the peritoneum [3, 4]. Third, MCS recapitulate aggressive tumorigenesis when transplanted into mice [5]. Fourth, ovarian cancer cells routinely propagating *in vitro* as MCS may generate a subpopulation of cancer stem/tumor-initiating cells, which are highly neoplastic [6–8]. These MCS are also particular critical in addressing the challenge of treating ovarian cancer in which current therapies are ineffective (5-year survival <25%). However, the factors that regulate MCS formation are largely unknown.

There is growing evidence supports the importance of p70 S6 kinase (p70^S6K^), a downstream effector of PI3K/Akt, in ovarian cancer [9]. p70^S6K^ activation occurs significantly more often in ovarian tumors than in benign or borderline lesions, and that constitutive activation of p70^S6K^ correlates with aggressive malignant phenotypes [10]. Although it was originally described as being predominantly involved in cell growth, we have provided the first evidence for a role of p70^S6K^ in other aspects of tumor progression, such as metastasis [10–12]. However, it is still not known whether p70^S6K^ affects MCS formation, although this is a key mechanism of ovarian cancer metastasis. The ribosomal protein S6 was the first and most well-known substrate of p70^S6K^. However, despite the diverse process controlled by p70^S6K^, only few substrates are known.

MicroRNAs (miRNAs) are small non-coding RNAs that have recently emerged as fundamental posttranscriptional regulators of target gene expression. Although first identified in *c. elegans*, miRNAs are now known to be expressed in most organisms and aberrant miRNA expression has been implicated in the pathogenicity of human cancers [13]. Of equal importance as transcriptional regulation, the processing of premature miRNAs is a critical rate-limiting step in miRNA biogenesis that controls mature miRNA turnover. Primary (pri-) miRNAs are processed into precursor (pre-) miRNAs by the dsRNA-specific ribonulease Drosha, whereas pre-miRNAs are cleaved to produce mature miRNAs by Dicer [14, 15]. While detailed insight has been gained into their target genes, little is known about signaling mechanisms controlling miRNA biogenesis, particularly those that relate to disease.

Here we show for the first time that p70^S6K^ is a pivotal regulator of MCS formation. We also provide mechanistic insight that a unique aspect of the function of p70^S6K^ by which it suppresses the maturation of miR-145, which controls the turnover of two critical transcripts Twist and Sox9 in the regulation of N-cadherin through tristetraprolin (TTP)/Dicer-dependent pathway.

## RESULTS

### p70^S6K^ promotes MCS formation

**Figure 1:**
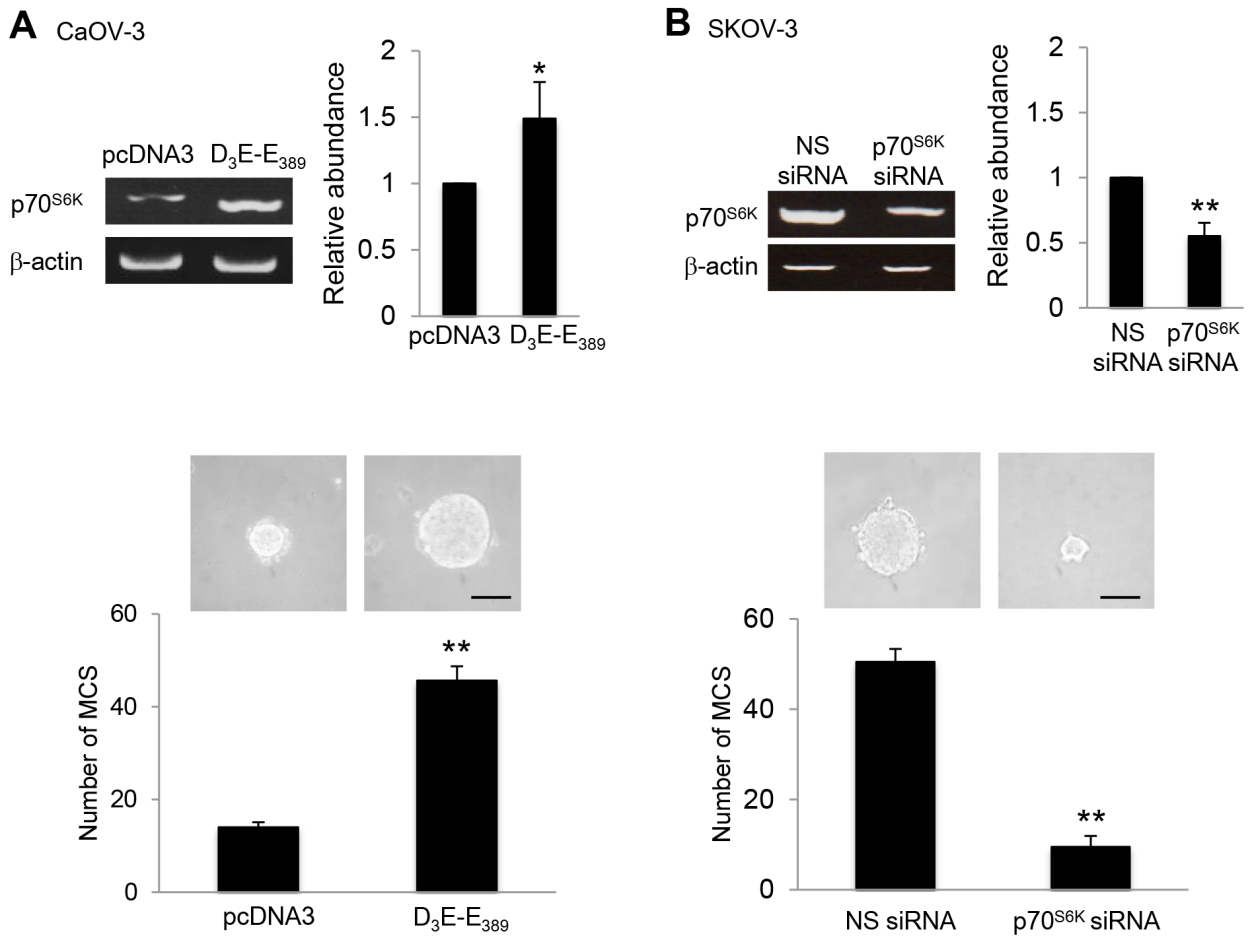
p70^S6K^ promotes MCS formation **A.** CaOV-3 cells were transfected with pcDNA3 or constitutively active p70^S6K^ construct (D_3_E-E_389_) or **B.** SKOV-3 cells were transfected with nonspecific (NS) siRNA or p70^S6K^ siRNA for 72 hr. Total RNA was extracted and reverse transcription-PCR was performed using p70^S6K^ sequence-specific primers. β-actin was included as a control. The band intensities were quantified by densitometric analysis and expression levels relative to that of β-actin were shown. The number of tumor spheres generated in CaOV-3 and SKOV-3 were photographed and counted. Bar = 100 μm. Results are presented as the mean ± SD and were analyzed using paired student's *t* test. *P < 0.05; **P < 0.005, compared with pcDNA3 or NS siRNA.

In addressing the factors that promote MCS formation, we considered the role of p70^S6K^, which is a key intracellular signaling mediator for the effects of multiple growth factors in the malignant ascites and is frequently hyperactive in human ovarian cancer [12]. Indeed, overexpression of the constitutively active p70^S6K^, (D_3_E-E_389_) was sufficient to robustly increase MCS formation (3.3-fold) (Figure 1A). Using a siRNA-based silencing approach, p70^S6K^ siRNA significantly suppressed MCS formation, establishing that p70^S6K^ as an endogenous promoter of MCS formation in ovarian cancer (Figure 1B). A second p70^S6K^ siRNA showed similar results (data not shown), and the nonspecific siRNA had no effect (Figure 1B).

### N-cadherin is the regulator of MCS phenotype upon p70^S6K^ activation

**Figure 2:**
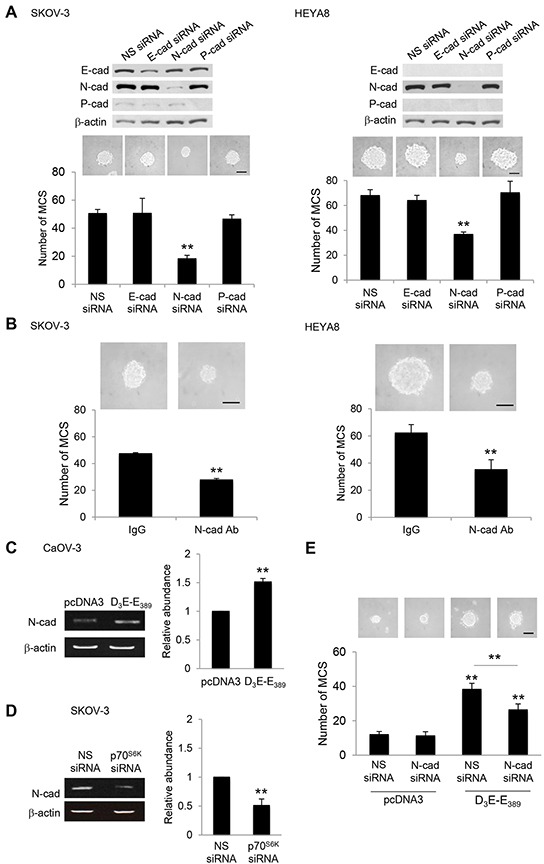
Silencing of N-cadherin abolishes MCS formation **A.** SKOV-3 or HEYA8 were transfected with nonspecific (NS) siRNA, E-cadherin siRNA, N-cadherin siRNA or P-cadherin siRNA. Expression of E-cadherin, N-cadherin and P-cadherin were assessed by Western blotting. β-actin was included as a loading control. The band intensities were quantified by densitometric analysis and expression levels relative to that of β-actin were indicated. The number of tumor spheres generated in were photographed and counted. **B.** SKOV-3 or HEYA8 cells were incubated with IgG or N-cadherin function blocking antibody for 72 hr. The number of tumor spheres generated was photographed and counted. **C.** CaOV-3 cells were transfected with pcDNA3 or constitutively active p70^S6K^ (D_3_E-E_389_) or **D.** SKOV-3 cells were transfected with NS siRNA or p70^S6K^ siRNA for 72 hr. Total RNA was extracted and reverse transcription-PCR was performed using N-cadherin sequence-specific primers. β-actin was included as a control. The band intensities were quantified by densitometric analysis and expression levels relative to that of β-actin are indicated. **E.** CaOV-3 cells were transfected with pcDNA3 or D_3_E-E_389_ in the presence of NS siRNA or N-cadherin siRNA for 72 hr. The number of tumor spheres generated was photographed and counted. Bar = 100 μm. Results are presented as the mean ± SD and were analyzed using paired student's *t* test. **P < 0.005, compared with NS siRNA, IgG or pcDNA3.

Cadherins are major mediators of cell-cell adhesion [16]. To begin addressing whether cadherin has a role in MCS formation, the expression of E-, N-, and P-cadherin, which have key roles in ovarian tumor progression [17] in relation to their ability to form MCS was first examined. Western blot analysis demonstrated that N-cadherin was highly expressed in SKOV-3 and HEYA8 cells, both of which have been shown to most effectively form compact spheroids (Supplementary Figure S1A & S1B). CaOV-3 and A2780 cells, which possess little or no N-cadherin, showed no MCS formation (Supplementary Figure S1A & S1B). Importantly, p70^S6K^ was expressed at higher levels in SKOV-3 and HEYA8 cells, which exhibit physiologically higher endogenous levels of N-cadherin (Supplementary Figure S1A & S1B). It is worth noting that this seems to be independent of the endogenous expression of E- or P-cadherin (Supplementary Figure S1A & S1B). Of note, depletion of N-cadherin by the use of siRNA, in which reduced the levels of N-cadherin, but not the levels of E- or P-cadherin, inhibited the ability of SKOV-3 and HEYA8 cells to form MCS (Figure 2A). No inhibition was observed for nonspecific siRNA, confirming that the effect is N-cadherin specific (Figure 2A). Similarly, hindering N-cadherin engagement with a function-blocking antibody also ablated MCS formation, whereas control IgG was without effect (Figure 2B). These results suggest that N-cadherin may have a dominant effect over other cadherins on MCS formation of ovarian cancer cells.

### p70^S6K^ increases N-cadherin expression via Twist and Sox9

To determine whether p70^S6K^ mediates MCS formation via N-cadherin, we first overexpressed constitutively active p70^S6K^, D_3_E-E_389_. This resulted in an effective upregulation of N-cadherin (Figure 2C). Conversely, knocking down p70^S6K^ clearly abolished the induction of N-cadherin (Figure 2D). Furthermore, inhibition of N-cadherin significantly suppressed MCS formation by D_3_E-E_389_ overexpression (Figure 2E), indicating also a functional correlation between p70^S6K^ and N-cadherin in MCS formation.

**Figure 3:**
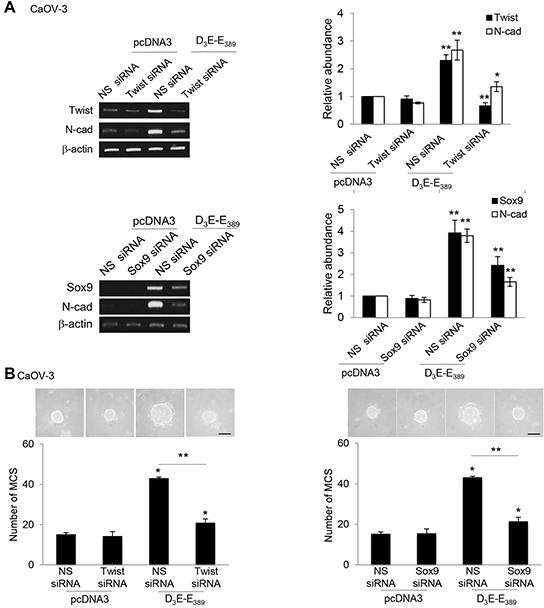
p70^S6K^ increases expression of Twist and Sox9 transcription factors **A.** CaOV-3 transfected with pcDNA3 or constitutively active p70^S6K^ (D_3_E-E_389_) in the presence of nonspecific (NS) siRNA or Twist siRNA or Sox9 for 72 hr. Total RNA was extracted and reverse transcription-PCR was performed using Twist, Sox9, and N-cadherin sequence-specific primers. The signal intensities were quantified by densitometric analysis and expression levels relative to that of β-actin are indicated. **B.** The number of tumor spheres generated was photographed and counted. Bar = 100 μm. Results are presented as the mean ± SD and were analysed using paired student's *t* test. *P < 0.05; **P < 0.005, compared with pcDNA3 or NS siRNA.

N-cadherin is known to be activated through at least two transcriptional regulators, including Twist and Sox9 [18]. This is also relevant to the clinical situation that Twist and Sox9 are often elevated in malignant ovarian tumors and confer a poor outcome to the late stage ovarian cancer patient survival [19, 20]. Given the robust effects of p70^S6K^ on N-cadherin, we investigated whether p70^S6K^ regulates expression of these two genes (Twist and Sox9). As shown in Figure 3A, D_3_E-E_389_ markedly induced an increase in Twist and Sox9 mRNA, which correlated with the upregulation of its target gene, N-cadherin (Figure 3A). In contrast, depletion of Twist and Sox9 through specific siRNAs led to repression of the D_3_E-E_389_-induced N-cadherin mRNA expression (Figure 3A). Furthermore, siRNA depletion of Twist and Sox9 inhibited MCS formation (Figure 3B), suggesting functional roles for them.

### p70^S6K^ activation stabilizes Twist and Sox9 mRNAs

**Figure 4:**
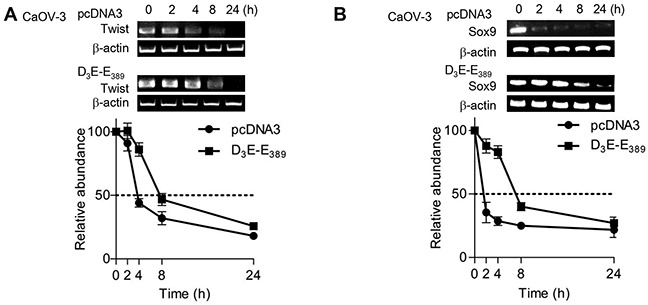
p70^S6K^ enhances Twist and Sox9 mRNA stabilities **A.** CaOV-3 cells transfected with pcDNA3 or D_3_E-E_389_ were incubated with actinomycin D (ActD; 5 μg/ml) over a time course of 0, 2, 4, 8 and 24 hr. Total RNA was then extracted and reverse transcription-PCR was performed using Twist and **B.** Sox9 sequence specific primers. β-actin was included as an internal control. Results are presented as the mean ± SD.

Since the increase of mRNA could result from either increased transcription or mRNA stability, this prompted us to investigate the cellular mechanisms underlying p70^S6K^ regulation of Twist and Sox9 mRNAs. Interestingly, D_3_E-E_389_ was without effect on the induction of luciferase activity driven by Twist- and Sox9-promoter regions (Supplementary Figure S2). We next performed RNA decay analyses using actinomycin D. Twist mRNA t_1/2_ was prolonged in D_3_E-E_389_-expressing CaOV-3 cells (t_1/2_ ~ 7.6 h) by approximately 2-fold (Figure 4A). D_3_E-E_389_ expression produced a similar stabilization of Sox9 mRNA (t_1/2_ ~ 7.5 h) (4.7-fold) (Figure 4B). These results suggest that p70^S6K^ may interfere with the mRNA degradation pathway to stabilize Twist and Sox9 mRNAs, leading to their accumulation.

### p70^S6K^ directly targets miR-145 to increase Twist and Sox9 expression

**Figure 5:**
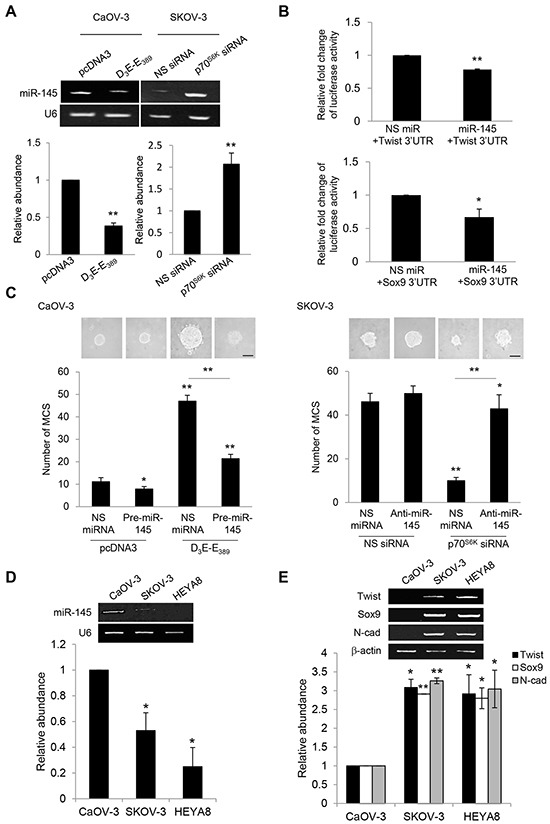
p70^S6K^ decreases Twist and Sox9 mRNA turnover via suppression of miR-145 **A.** CaOV-3 cells were transfected with pcDNA3 or constitutively active p70^S6K^ (D_3_E-E_389_) or SKOV-3 cells were transfected with nonspecific (NS) siRNA or p70^S6K^ siRNA for 72 hr. Total RNA was extracted and reverse transcription-PCR was performed using mature miR-145 sequence-specific primers. U6 was included as an internal control. The signal intensities were quantified by densitometric analysis and the amounts were normalized for the amount of U6. **B.** CaOV-3 cells were transiently transfected with 0.5 μg of the luciferase reporter gene fused with Twist 3′UTR or Sox9 3′UTR and 15 ng of β-galactosidase plasmid for 72 hr. Luciferase and β-galactosidase activities were assayed, and the luciferase activity of each sample was normalized with β-galactosidase activity. **C.** CaOV-3 cells were transfected with pcDNA3 or D_3_E-E_389_ in the presence of NS miRNA or precursor miR-145 (pre-miR-145) or SKOV-3 were transfected with NS siRNA or p70^S6K^ siRNA in the presence of NS miRNA or miR-145 inhibitor (anti-miR-145) for 72 hr. The number of tumor spheres generated was photographed and counted. Bar = 100 μm. **D.** CaOV-3, SKOV-3 and HEYA8 were cultured in non-adherent culture dish for 72 hr. Total RNA was extracted and reverse transcription-PCR was performed using sequence-specific primers to mature miR-145 (mat-miR-145). U6 was included as an internal control. **E.** Twist, Sox9, and N-cadherin sequence-specific primers. β-actin was included as an internal control. The signal intensities were quantified by densitometric analysis and the amounts were normalized for the amount of U6 or β-actin. Results are presented as the mean ± SD and were analyzed using paired student's *t* test. *P < 0.05; **P < 0.005, compared with NS siRNA, pcDNA3 or NS miRNA.

Given the importance of p70^S6K^ in mediating Twist and Sox9 mRNA half-lives, we were particularly interested in miRNAs that have been demonstrated to function in these processes [21]. To address this question, we employed a systematic approach to identify such miRNA regulators which should have the ability to bind the Twist and Sox9 mRNAs. We first compared the set of miRNAs predicted to target Twist and/or Sox9 3′UTR using miRNAmap 2.0 which employs a variety of prediction databases (Target Scan program, miRanda algorithm, and RNAhybrid program). The analysis, followed by examination of the set of miRNAs known to be associated with ovarian cancer progression [22–24], revealed 17 miRNAs (let-7b, miR-10b, miR-105, miR-127, miR-133a, miR-145, miR-147, miR-154, miR-199a, miR-199b, miR-214, miR-299, miR-302c, miR-331, miR-337, miR-376, miR-424) to target either Twist and/or Sox9 mRNAs and also present at lower levels in the metastatic progression of ovarian cancer. Among these miRNAs, interestingly, only the expression of miR-145 was significantly repressed by the constitutively active p70^S6K^ and increased by the p70^S6K^ siRNA as compared to their respective empty vector and nonspecific siRNA controls (Figure 5A). These results suggest that p70^S6K^ promotes the expression of specific miRNAs.

In order to verify whether Twist and Sox9 are directly targeted by miR-145, we examined the effect of miR-145 on the expression of Twist and Sox9 through heterologous luciferase reporter assays. As shown, miR-145 was able to repress the luciferase activity of reporter containing the 3′ untranslated regions (UTR) of both Twist and Sox9, revealing Twist and Sox9 to be directly targeted by miR-145 (Figure 5B). Consistent with the phenotypes of its target genes, addition of pre-miR-145 abrogated D_3_E-E_389_-mediated MCS formation, whereas addition of anti-miR-145 reversed the suppression of MCS formation resulting from p70^S6K^ knockdown, indicating that miR-145 is necessary for the promotion of MCS (Figure 5C). To investigate whether the potential regulation of miR-145 on Twist and Sox9 also correlated with MCS formation potential, we compared the levels of miR-145 with the expression levels of Twist and Sox9. We observed that SKOV-3 and HEYA8 showing little or no miR-145 also expressed high levels of Twist and Sox9 (Figure 5D). Expression of N-cadherin which determines the ability to form MCS was significantly more frequent in SKOV-3 and HEYA8 than CaOV-3 (Figure 5E).

### A specific role for Dicer microprocessor in miR-145 turnover

**Figure 6:**
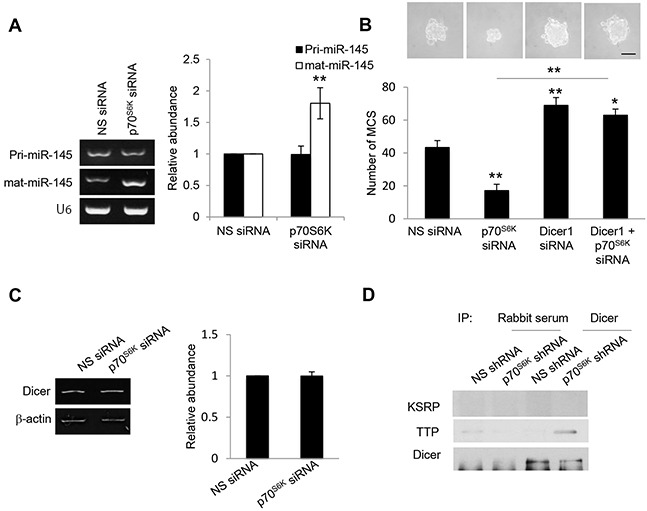
p70^S6K^ downregulates mature miR-145 but not primary miR-145 **A.** SKOV-3 cells were transfected with nonspecific (NS) siRNA or p70^S6K^ siRNA for 72 hr. Total RNA was extracted and reverse transcription-PCR was performed using sequence-specific primers to primary miR-145 (pri-miR-145) and mature miR-145 (mat-miR-145). U6 was included as an internal control. The signal intensities were quantified by densitometric analysis and the amounts were normalized for the amount of U6. **B.** SKOV-3 cells were transfected with NS siRNA, p70^S6K^ siRNA, or Dicer siRNA for 72 hr. The number of tumor spheres generated was photographed and counted. **C.** SKOV-3 cells were transfected with nonspecific (NS) siRNA or p70^S6K^ siRNA for 72 hr. Total RNA was extracted and reverse transcription-PCR was performed using Dicer sequence-specific primers. β-actin was included as an internal control. The signal intensities were quantified by densitometric analysis and expression levels relative to that of β-actin are indicated. **D.** p70^S6K^ siRNA-transfected SKOV-3 cells were immunoprecipitated with Dicer antibody. Rabbit serum was used as a control. The immunocomplex was resolved using Western blotting and detected with KSRP and TTP antibodies. Bar = 100 μm. Results are presented as the mean ± SD and were analyzed using paired student's *t* test. *P < 0.05; **P < 0.005, compared with NS siRNA.

To determine how p70^S6K^ regulates miR-145 expression, we assessed different aspects of miR-145 expression in response to p70^S6K^. Despite the increases in mature miR-145 expression, no significant changes were observed in primary miR-145 levels following p70^S6K^ siRNA as determined by RT-PCR (Figure 6A). Furthermore, it has also been shown that its downregulation observed in various tumors is not due to DNA copy number loss [24, 25], suggesting that p70^S6K^ may post-transcriptionally regulate miR-145 expression. One post-transcriptional mechanism by which p70^S6K^ could possibly downregulate the levels of mature miR-145 is by suppressing the maturation of miR-145. Previous reports have demonstrated that Dicer serves as a major regulator of the miRNA biogenesis machinery [26]. Knocking down Dicer clearly abolished the p70^S6K^ siRNA-mediated suppression of MCS formation (Figure 6B), suggesting a novel link between p70^S6K^ and Dicer in MCS formation and warrants a closer examination into the mechanism through which p70^S6K^ regulates Dicer.

### p70^S6K^ impairs the interaction of TTP with Dicer

We first examined the potential regulation of p70^S6K^ on Dicer expression, but found no effect (Figure 6C), suggesting that p70^S6K^ may regulate Dicer via its interacting partners. The recruitment of AU-rich RNA-binding protein (ARE-BP), KH-type splicing regulatory protein (KSRP), to Dicer is critical for loading miRNA precursors onto the microprocessor complex and facilitating miRNA maturation from precursor to mature miRNAs [27]. However, we found no similar interaction between KSRP and Dicer upon p70^S6K^ activation (Figure 6D). Thus we investigated whether other members of ARE-BPs may physically associate with Dicer. TTP displayed lower levels in the metastatic progression of ovarian cancer [28] albeit previously not known to interact with Dicer. Coimmunoprecipitation experiments showed that silencing of p70^S6K^ significantly enhanced the interaction of TTP with Dicer (Figure 6D), suggesting that TTP can bind Dicer.

### The miRNA destabilizing factor TTP is phosphorylated by p70^S6K^

**Figure 7:**
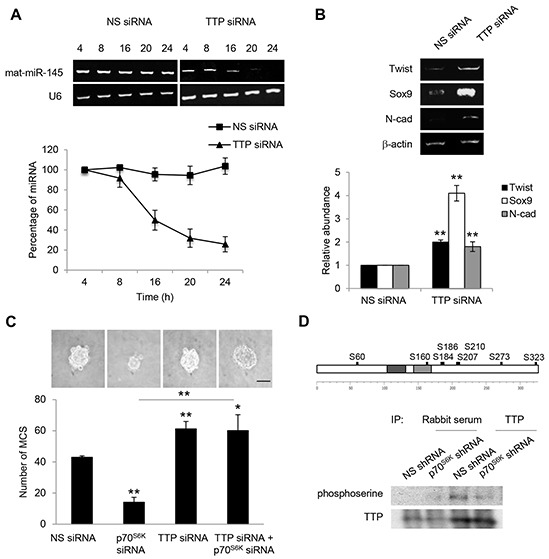
TTP increases the level of mature miR-145 **A.** SKOV-3 cells were transfected with nonspecific (NS) siRNA or TTP siRNA over a time course of 4, 8, 16, 20 and 24 hr. Total RNA was extracted and reverse transcription-PCR was performed using sequence-specific primers to mature miR-145 (mat-miR-145). U6 was included as an internal control. The signal intensities were quantified by densitometric analysis and the amounts were normalized for the amount of U6. **B.** Reverse transcription-PCR was also performed using sequence-specific primers to Twist, Sox9 and N-cadherin. β-actin was included as an internal control. The signal intensities were quantified by densitometric analysis and expression levels relative to that of β-actin are indicated. **C.** SKOV-3 cells were transfected with NS siRNA, p70^S6K^ siRNA or TTP siRNA for 72 hr. The number of tumor spheres generated was photographed and counted. **D.** *In silico* analysis of the TTP phosphorylation sites were performed using the Human Phosphosite Knowledgebase available at PhosphoNet (http://www.phosphonet.ca/). Phosphorylation sites with a Kinase Predictor score greater or equal to 350 were indicated. p70^S6K^-stable knockdown SKOV-3 cells were immunoprecipitated with TTP antibody. Rabbit serum was used as a control. The immunocomplexes were resolved using Western blotting and detected by a phosphoserine antibody. Bar = 100 μm. Results are presented as the mean ± SD and were analyzed using paired student's *t* test. *P < 0.05; **P < 0.005, compared with NS siRNA or p70^S6K^ siRNA.

Knockdown of TTP obtained using siRNA led to a more than 75% reduction in the steady-state level of miR-145 (Figure 7A). Furthermore, Twist, Sox9, and N-cadherin mRNAs were stable in TTP siRNA-transfected cells (Figure 7B). Importantly, knockdown of TTP significantly restored the decreased MCS formation mediated by knockdown of p70^S6K^ (Figure 7C). Together, these results indicate that TTP is crucial in controlling miR-145 maturation and, in turn, Twist/Sox9-mediated N-cadherin expression and MCS formation. The phosphorylation of RNA-binding proteins modulates their association with Dicer [29]. We further investigated whether p70^S6K^ was able to phosphorylate TTP. Using PhosphoNet (www.Phosphonet.ca/), a kinase substrate prediction database, TTP has been found to be a putative substrate for p70^S6K^ (Figure 7D). Knockdown of p70^S6K^ significantly reduced the phosphorylation of TTP while not empty vector control, as shown by anti-TTP immunoprecipitation following phospho-serine detection (Figure 7D), suggesting that p70^S6K^ is a novel upstream regulator of the TTP.

### p70^S6K^ and its associated signaling in human ovarian carcinoma

**Figure 8:**
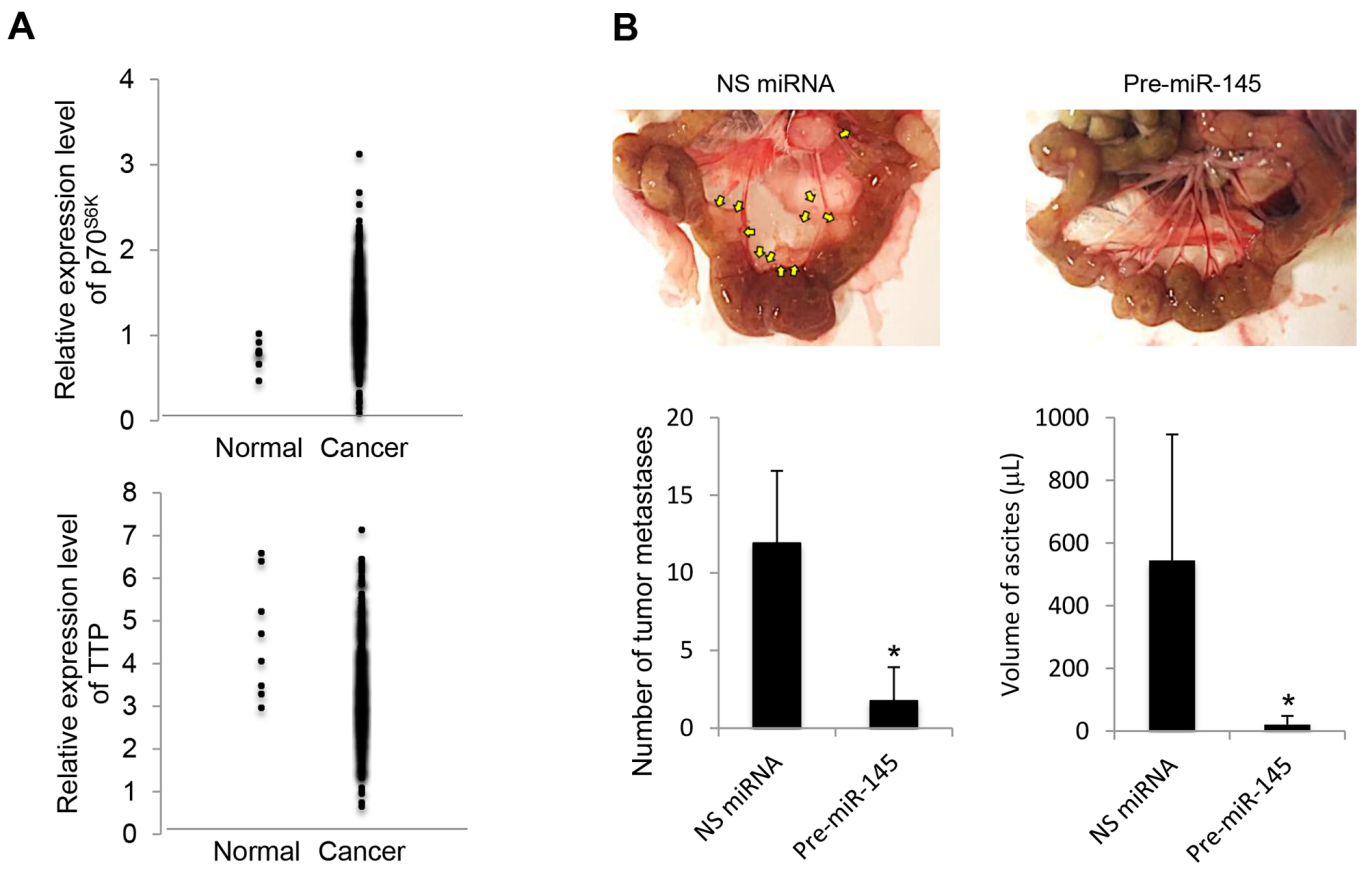
p70^S6K^ and TTP expression in human ovarian cancers and miR-145 upregulation results in decreased cancer progression *in vivo* **A.** Clinical data were retrieved from the TCGA Ovarian dataset which contains 594 samples available on Oncomine (https://www.oncomine.org). **B.** NOD/SCID mice were intraperitoneally injected with SKOV-3 cells transduced by nonspecific (NS) miRNA or precursor miR-145 (pre-miR-145). At the time of sacrifice, the peritoneal cavity was assessed for evidence of metastases. The metastatic lesions were excised and their total number was counted, and ascites volume was measured. Results are presented as the mean ± SD. *P < 0.05 compared with NS miRNA.

To understand more about the clinical relationship between p70^S6K^ and TTP levels in ovarian tumors, we used the publicly available datasets from Oncomine (available at: https://www.oncomine.org) to survey a large number of ovarian cancers. There was a significant inverse association between p70^S6K^ and TTP levels, in which p70^S6K^ was highly expressed in tumor samples whereas TTP was suppressed (Figure 8A). Next, we transplanted nonspecific miRNA and precursor miR-145 MCS cells into the peritoneum of NOD/SCID mice to determine whether miR-145 activation actually produced a less malignant phenotype *in vivo*. The results showed that highly aggressive tumors with visible tumor masses growing on the omentum, mesenteries, and small bowels with developed ascites reflecting characteristics commonly displayed by ovarian cancer lesions were observed in mice transplanted with nonspecific miRNA (Figure 8B). In contrast, the number and the size of tumor nodules and ascites volume were substantially decreased by treatment with precursor miR-145 (Figure 8B). These studies suggest that p70^S6K^ and its associated signaling may represent useful targets for the treatment of aggressive cell characteristics of ovarian cancer *in vivo*.

## DISCUSSION

Metastatic progression requires effectors proteins associated with the same cellular phenotype to be coherently expressed. In recent years, posttranscriptional regulation has emerged as a robust regulation of coordinated gene expression and phenotype manifestation. The most studied of which is small noncoding miRNAs. In this study, we show for the first time that p70^S6K^, which is frequently hyperactive in human ovarian cancer [11], as a novel component of the miRNA biogenesis machinery, which governs post-transcriptional maturation of a subset of miRNAs, and that this regulation is important in effecting MCS formation.

As a key kinase in the control of target mRNA translation, p70^S6K^ is logically considered to be a modulator of protein synthesis. Here we provide the first demonstration that p70^S6K^, a key kinase in the control of target mRNA translation, can regulate miRNA biogenesis. This role of p70^S6K^ in the miRNA-generating complex regulation is one of the most important and exciting emerging paradigms, which may link its multiple facets of cell activities seen [10], and its therapeutic inhibition displays *in vivo* efficacy highlights the significance of p70^S6K^ as a key promoter of ovarian metastatic progression.

The regulation of miRNA remains still largely unknown. Clearly, transcriptional regulation is an essential control. Alternatively, the posttranslational modification of miRNAs, albeit less studied, has recently garnered much attention as an important regulatory mechanism of miRNA activities. Various kinases, such as p38 MAPK, Akt, and ATM have been shown to phosphorylate KSRP of the miRNA-generating complex at different residues and regulate its biological activities [30, 31]. This study is the first to demonstrate that TTP, an ARE-BP that has been widely studied for posttranscriptional gene regulation and miRNA biogenesis [27], is a substrate of p70^S6K^, adding p70^S6K^ as a new member of posttranscriptional modulators of miRNAs, and TTP as a new protein interactor of Dicer. In accordance with our findings, while changes in the expression level of Dicer was not frequently observed in ovarian cancer tissues [24], TTP loss is found in many types of cancers including ovarian cancer and this downregulation correlates with increased metastatic potential and poor prognosis [28, 32–34]. Although it is possible that TTP may disrupt the global translation of all miRNAs, our existing data suggest that not all miRNAs are targets of TTP. Likewise, a G-rich motif in precursor miRNAs has been identified as a critical regulatory element in KSRP, an ARE-BP-mediated miRNA maturation, suggesting regulation specificity of ARE-BP on miRNA maturation [35].

Little is known about the regulation of N-cadherin. Here we show for the first time that miR-145 divergently targets Twist and Sox9 and comprise a coordinated network that maximally enhances N-cadherin signaling. Consistently, seeding regions of miR-145 has been identified in 461-488 of the Twist 3′UTR (NG_008114.1) and 265-289 of the Sox9 3′UTR (NG_012490.1). The simultaneous targeting of multiple genes for metastasis promotion by a single metastasis suppressor miRNA may explain the dramatic effects observed [36], highlighting the significance of miR-145 as a key tumor suppressor of ovarian cancer progression. Indeed, miR-145 is one of the most frequently studied miRNAs detected in human cancers, and it has been validated to be downregulated in ovarian cancer [22]. miR-145 downregulation is associated with ovarian cancer progression and that low miR-145 expression correlates with poor survival [37]. It is also relevant to the clinical situation that Twist and Sox9 are often elevated in malignant ovarian tumors and Twist expression confers a poor outcome to the late stage ovarian cancer patient survival [19, 20].

The importance of miRNAs in cancer biology has opened avenues for drug development based on the inhibition of oncomiRs or replacement of suppressor miRNAs [38, 39]. The advantage of using miRNA approaches is based on its ability to simultaneously target multiple genes that may be involved in coregulating a given function. Traditional gene therapy is hindered by inefficient delivery of large encoding gene into the nucleus. On the contrary, miRNAs are small in size and active in cytoplasm [40]. Liposome-based nano-sized carrier-mediated miRNA delivery constitutes a promising nanomedicine approach in cancer therapy. Indeed, the safety and efficacy of miR-34 liposomal injection is undergoing Phase I clinical trials on liver, lung, renal cell carcinomas, and other hematologic malignancies (NCT01829971, https://clinicaltrials.gov/ct2/show/NCT01829971?term=NCT01829971&rank=1). The closed space of the peritoneal cavity may offer an advantage for gene transfer in that the therapeutic genes can be sensitive and specific to the target cells.

Deregulation of p70^S6K^ signaling frequently occurs in many human cancers, and it appears to play an important role in their progression. For example, active p70^S6K^-expressing breast cancer has a worse prognosis [41]. Furthermore, p70^S6K^ overexpression in breast, colon, liver, and ovarian tumors are found to be related to aggressive malignant phenotypes [11, 42–44], suggesting that the role of p70^S6K^ in MCS that we propose in ovarian cancer may be extended to other tumor cell types and thereby p70^S6K^ could be an attractive target for therapeutics. Despite the fact that many PI3K pathway inhibitors have been developed, the clinical trials with these drugs have not been promising due to crosstalk with multiple pathways and the presence of potent feedback loops [45]. Targeting p70^S6K^ may thus represent a potential therapeutic advantage over current PI3K inhibitors, which might prove to be more effective and specific while minimizing toxicity. Moreover, the addiction of many tumors to the pathway provides a rationale for PI3K/Akt target, particularly p70^S6K^, exploitation.

In summary, we have established a novel regulatory link between p70^S6K^ and miRNA biogenesis that not only highlights a new mechanism through which p70^S6K^ functions to establish its pro-tumor regimen but also defines the MCS properties. These findings introduce a new dimension to our existing knowledge of the regulation of miRNA machinery and signaling molecules that remains to be explored by which can be exploited as more precise targets for therapy. The prominent role of p70^S6K^ in a broad range of human cancers suggests that its targeting may have key roles, not only in ovarian cancer, but also in other tumors.

## MATERIALS AND METHODS

### Cell culture and treatments

The human epithelial ovarian cancer cell lines SKOV-3, CaOV-3, and A2780 were gifts from Dr. N. Auersperg (University of British Columbia, Vancouver, B. C., Canada). HEYA8 was a gift from Dr. J. Liu (MD Anderson Cancer Center, Houston, TX). Cells were routinely grown in medium 199:MCDB 105 (1:1) (Sigma, St. Louis, MO) with 10% fetal bovine serum (Hyclone, Logan, UT) and 1% penicillin-streptomycin mixture (Invitrogen, Carlsbad, CA) in a humidified 95% air, 5% CO_2_ incubator at 37°C before experiments. MCS were generated by liquid overlay method [46]. Briefly, 5,000 cells/ml were plated into dishes coated with 0.5% agarose (Invitrogen, Carlsbad, CA) (non-adherent culture conditions) for 72 h after treatment. The number of MCS generated with ≧100 μm diameter was counted. Mature miR-145 mimic were purchased from Ambion (Austin, TX). Synthetic oligos, mock with no homology to human genome, were used as a negative control. p70^S6K^ siRNA, Twist siRNA, Sox9 siRNA were acquired from Dharmacon (Lafayette, CO). miRNA or siRNA duplex were transfected into cells by using Lipofectamine 2000 according to the manufacturer's protocol (Invitrogen, Carlsbad, CA).

### Measurement of mRNA stability

The half-life of Twist and Sox9 mRNAs were determined using actinomycin D chase experiments, following a standard protocol described elsewhere [47]. Briefly, actinomycin D was added to a final concentration of 5 μg/ml to block further transcription. At 0, 2, 4, 8, and 24 hr after actinomycin D treatment, the cells were harvested, and mRNA was quantified by PCR as described above. The percentages of mRNA over time before and after the addition of actinomycin D were determined.

### Reverse transcription-polymerase chain reaction

Total RNA was isolated by Trizol (Invitrogen, Carlsbad, CA). For detecting miR-145 levels, RNA was reversed transcribed to cDNA by TaqMan miRNA assays (Applied Biosystems, Foster City, CA) using specific stem-loop reverse transcription primer (5′-GTTGGCTCTGGTGCAGGGTCCGAGGTATTCGCACCAGAGCCAACAGGAT-3). The expression levels of mature miR-145 was quantified by PCR using sequence specific primers (Forward: 5′-CGGCAGGTCAAAAGGGTCCT-3′; Reverse: 5′-TG CAGG GTCCGAGGTATTCG-3′). Endogenous U6 small nuclear RNA levels were determined simultaneously for normalization.

### miRNA target prediction

To define potential upstream regulators of Twist and Sox9, we matched candidate miRNAs that were predicted by using miRNAMap 2.0 (available at http://mirnamap.mbc.nctu.edu.tw/) which employs 3 publicly available algorithms, miRanda (http://www.microrna.org/microrna/home.do), TargetScan (http://www. targetscan.org/), and RNA hybrid (http://bibiserv.techfak.uni-bielefeld.de/rnahybrid).

### Western blot analysis

Cell lysates (20 μg protein each) were separated by 7.5% SDS-polyacrylamide gel electrophoresis and transblotted onto nitrocellulose membrane (Bio-Rad). The membrane was blocked with 5% nonfat dry milk and incubated with anti-phospho-p70^S6K^ (Thr389) (1:1,000) (Cell Signaling), anti-Twist (1:1,000) (Santa Cruz), anti-Sox9 (1:1,000) (Abcam), anti-N-cadherin (1:1,000) (Zymed), anti-E-cadherin (1:1,000) (BD Transduction Laboratories) in phosphate-buffered saline containing 0.1% Tween 20 rotating at 4°C overnight. After washing, the membrane was further incubated with secondary antibodies coupled to horseradish peroxidase at room temperature for 1 h and developed with an enhanced chemiluminescent detection kit (Amersham).

### Luciferase reporter assay

The putative miR-145 binding sites at the 3′UTR of Twist and Sox9 were cloned downstream of a cytomegalovirus promoter-driven firefly luciferase cassette in a vector. The β-galactosidase plasmid was cotransfected as internal control in the presence of either synthetic miR-145 or mock control. Luciferase reporter assay (Promega, Madison, WI) was performed 72 hr after transfection.

### *In vivo* intraperitoneal metastasis model

All animal care and experimental procedures were carried out according to the Committee for the Use of Laboratory Animals of the University of Hong Kong approved protocols. SKOV-3 MCS (2 × 10^5^ cells) stably transduced with nonspecific miRNA or pre-miR-145 were injected intraperitoneally into 6- to 8-week-old female NOD/SCID mice (Charles River Laboratories, Wilmington, MA) (*n* = 3 mice per group, and the experiment was conducted twice). Mice were assessed weekly for signs of pain and discomfort, weight change, and ascites formation. At the time of sacrifice, the number of all visible (> 0.1 cm diameter) tumor nodules in the peritoneal cavity and the volume of ascites were assessed as evidence of metastases.

### Statistical analysis

All experiments were carried out in triplicates and repeated at least two times with each experiment yielding essentially identical results. Data are presented as mean ± S.D. Student's t-test and analysis of variance was performed in GraphPad Prism (GraphPad, San Diego, CA) to determine differences between the experimental groups and the control groups. A *P*-value less than 0.05 was regarded as statistically significant.

## SUPPLEMENTARY FIGURES


