# Supplementary material for: A novel p70 S6 kinase-microRNA biogenesis axis mediates multicellular spheroid formation in ovarian cancer progression
Source: Oncotarget. 2016 May 13;7(25):38064–77. doi: 10.18632/oncotarget.9345 (PMC5122372; doi:10.18632/oncotarget.9345)
Supplement: Supplementary file 1 [file oncotarget-07-38064-s001.pdf]

SUPPLEMENTARY FIGURES

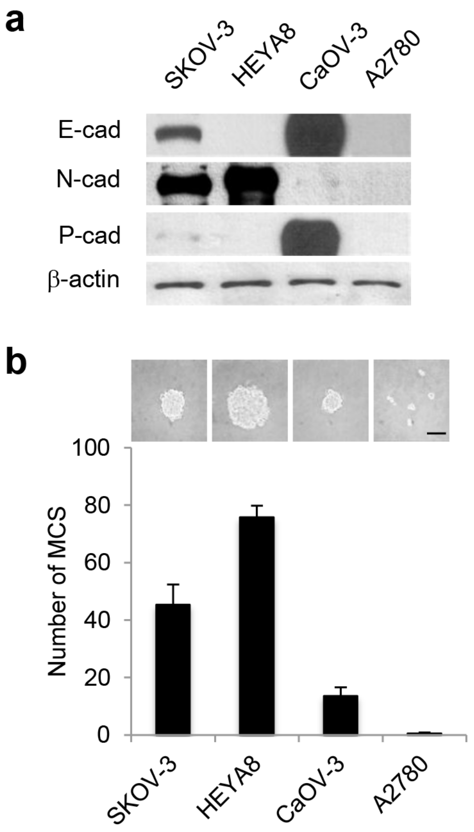

Supplementary Figure S1: N-cadherin-expressing cells have higher MCS formation ability. A. Expression of E-cadherin, N-cadherin and P-cadherin in SKOV-3, HEYA8, CaOV-3 and A2780 were analyzed using Western blot. β-actin was included as a loading control. **B.** SKOV-3, HEYA8, CaOV-3 and A2780 were cultured in non-adherent culture dish for 72 hr. The number of tumor spheres generated was photographed and counted. Bar = 100 µm. Results are presented as the mean ± SD.

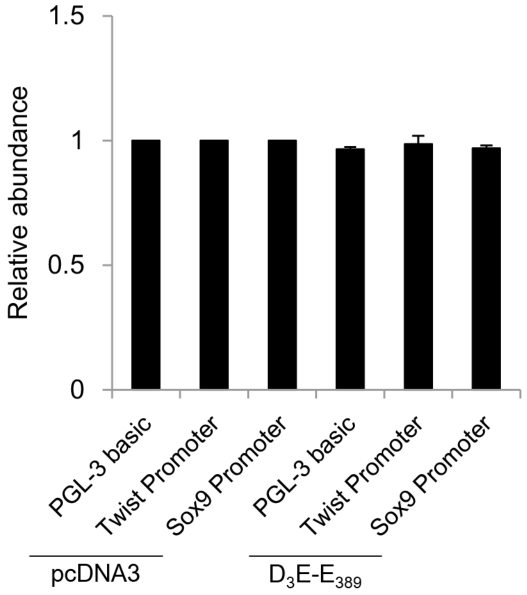

Supplementary Figure S2: p70^{S6K} has no effect on Twist and Sox9 promoter. CaOV-3 cells were transiently transfected with 0.5 µg of the luciferase promoter reporter gene of Twist or Sox9 and 15 ng of β-galactosidase plasmid and cultured in non-adherent culture dish for 72 hr. Luciferase and β-galactosidase activities were assayed, and the luciferase activity of each sample was normalized with β-galactosidase activity. Results are presented as the mean ± SD and were analyzed using Paired student's *t* test.
